# Oral corticosteroid use, morbidity and mortality in asthma: A nationwide prospective cohort study in Sweden

**DOI:** 10.1111/all.13874

**Published:** 2019-06-11

**Authors:** Magnus Ekström, Bright I. Nwaru, Pål Hasvold, Fredrik Wiklund, Gunilla Telg, Christer Janson

**Affiliations:** ^1^ Department of Clinical Sciences Lund, Respiratory Medicine and Allergology, Faculty of Medicine Lund University Lund Sweden; ^2^ Krefting Research Centre, Institute of Medicine University of Gothenburg Gothenburg Sweden; ^3^ Wallenberg Centre for Molecular and Translational Medicine University of Gothenburg, Uppsala University Uppsala Sweden; ^4^ AstraZeneca Nordic‐Baltic Södertälje Sweden; ^5^ Statisticon AB Uppsala Sweden; ^6^ Department of Medical Sciences: Respiratory, Allergy and Sleep Research Uppsala University Uppsala Sweden

**Keywords:** asthma, corticosteroids, morbidity, mortality

## Abstract

**Background:**

Patterns and determinants of long‐term oral corticosteroid (OCS) use in asthma and related morbidity and mortality are not well‐described. In a nationwide asthma cohort in Sweden, we evaluated the patterns and determinants of OCS use and risks of OCS‐related morbidities and mortality.

**Methods:**

Data for 217 993 asthma patients (aged ≥ 6 years) in secondary care were identified between 2007 and 2014 using Swedish national health registries. OCS use at baseline was categorized: regular users (≥5 mg/d/y; n = 3299; 1.5%); periodic users (>0 but <5 mg/d/y; n = 49 930; 22.9%); and nonusers (0 mg/d/y; n = 164 765; 75.6%). Relative risks of becoming a regular OCS user and for morbidity and mortality were analysed using multivariable Cox regression.

**Results:**

At baseline, 24% of asthma patients had used OCS during the last year and 1.5% were regular users. Of those not using OCS at baseline, 26% collected at least one OCS prescription and 1.3% became regular OCS users for at least 1 year during the median follow‐up of 5.3 years. Age at asthma diagnosis, increasing GINA severity and Charlson Comorbidity Index were associated with regular OCS use. Compared to periodic and non‐OCS use, regular use was associated with increased incidence of OCS‐related morbidities and greater all‐cause mortality, adjusted HR 1.34 (95% CI 1.24‐1.45).

**Conclusions:**

Oral corticosteroids use is frequent for asthma patients, and many are regular users. Regular OCS use is associated with increased risk of morbidity and mortality. These findings indicate that there is a need of other treatment options for patients with severe asthma who are using regular OCS.

## Graphical abstract

Annually, almost one in seven asthma patients used oral corticosteroids, which was stable over the 10‐year study period. Oral corticosteroids are still a substantial part of current asthma management for many patients and are associated with severe side effects and mortality risk. There is a need for other treatments for severe asthma patients who are using regular oral corticosteroids.

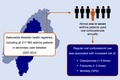

## 1 INTRODUCTION

Asthma is a common chronic disease affecting approximately 339 million people worldwide.^1^ Management of the disease is constantly evolving, with new available treatment options and updated international guidelines.^2^ The majority of asthma patients have mild or moderate disease, of which most are successfully managed.^2^ However, of the 4%‐8% of patients with severe asthma in the Nordic countries, many still live with a poorly controlled disease affecting everyday life.^3^, ^4^ A substantial percentage of severe asthma patients need treatment with oral corticosteroids (OCS), both as short‐term treatment for exacerbations, and as a regular long‐term maintenance treatment for asthma control.^3^ Regular OCS use is associated with both short‐ and long‐term OCS‐related complications, such as osteoporosis, fractures, ischaemic heart disease, hypertension and changes in glucose metabolism.^5^, ^6^, ^7^, ^8^, ^9^, ^10^

Whilst regular OCS use still plays a significant part in the treatment of patients with severe asthma, few population studies have to date investigated the patterns and determinants of long‐term OCS use, changes in treatment patterns over time and the impact of OCS use on morbidity and mortality. This may be due to limited number of countries with access to complete nationwide data. Sweden, with its nationwide, longitudinal and mandatory registers is an ideal setting to study OCS treatment in asthma from a whole population perspective.^11^ This enables inclusion of all patients in the country diagnosed with asthma in a secondary care setting, with linkage to near complete data on all collected medications from pharmacies, diagnosed diseases and causes of death.

The aim of this study was to evaluate the patterns of OCS use and its potential determinants, and the associations between OCS use and the risk of OCS‐related morbidities and all‐cause and cause‐specific mortality for asthma patients diagnosed in secondary care.

## 2 METHODS

### 2.1 Study design and data sources

This was a prospective, longitudinal, observational cohort study utilizing national Swedish health registries: (a) The National Patient Register (NPR) covering all hospital admissions since 1987 and outpatient specialist visits since 2001; (b) the Prescribed Drug Registry, covering all collected outpatient drug prescriptions since July 2005 using the anatomical Therapeutic Chemical (ATC) codes; and (c) the Cause of Death Register.^11^, ^12^, ^13^ Individual patient data were linked by the Swedish National Board of Health and Welfare using each person’s unique social security number. The study protocol was approved by the Stockholm Regional Ethics Committee (registration number 2017/4:2).^14^, ^15^

### 2.2 Study population

The study population included all asthma patients between 12 and 45 years in Sweden during 2007‐2014 in outpatient or inpatient secondary care, and a drug claim for obstructive lung disease (ATC R03). A defined index date and baseline period was utilized to classify patients into different OCS usage groups. The index date was the date of first asthma diagnosis and the baseline period was from the index date up to 365 days post index.

The following exclusion criteria were used:

- asthma diagnosis only before the age of 6 years,
- patients with <365 days of follow‐up from index date,
- drug claim of oral glucocorticoid other than prednisolone (ATC H02AB06) and betamethasone (ATC H2AB01) any time prior to index date or during the baseline period.

The reason for this is that prednisolone and betamethasone are the only OCS recommended for treatment of asthma in the Swedish Asthma Guidelines.^16^

Use of OCS equivalent to >20 mg/d of prednisolone during the full 1‐year baseline period as a higher dose would typically be used for conditions other than asthma.

Patients with other conditions for which systemic corticosteroids may be prescribed (ie, Crohn’s disease [K50], ulcerative colitis [K51], rheumatoid arthritis [M05], emphysema [J43], chronic obstructive pulmonary disease [J44], bronchiectasis [J47], cystic fibrosis [E84] and current malignancy [C00‐97]) were excluded.

Medical history data were retrieved from the NPR by ICD‐10 codes during 10 years prior to the date of asthma diagnosis and during the baseline period, except for current malignancies which was retrieved within 12 months prior asthma diagnosis and during the baseline period.

### 2.3 Study measures

#### 2.3.1 Oral corticosteroids use

Oral corticosteroids use during the baseline period was classified using the collected mean daily dosage per year (prednisolone equivalent) of prednisolone or betamethasone: regular users (OCS equivalent to an average ≥5 mg/d/y, corresponding to cumulative dosage ≥1825 mg prednisolone within 1 year); periodic users (OCS equivalent averaging more than zero but <5 mg/d/y); and nonusers (OCS equivalent to 0 mg/d/y). During follow‐up, OCS nonusers at baseline who subsequently collected OCS were further classified as regular OCS users (average ≥ 5 mg/d/y); ≥1 OCS collected; and patients dispensing ≥2 OCS.

#### 2.3.2 Determinants of oral corticosteroids use

Data on potential determinants of OCS use was collected at index date: age, sex, severity of asthma (Global Initiative for Asthma [GINA] step) and Charlson Comorbidity Index.^2^, ^17^

#### 2.3.3 Follow‐up time

Patients were followed prospectively for OCS use and death from the first day after the baseline period until date of death, emigration, dispense of OCS other than prednisolone and betamethasone, date of diagnosis of conditions for which systemic corticosteroids may be prescribed (see exclusions criteria), malignancies or end of follow‐up (31 December 2016), whichever occurred first. Assessment of comorbidity and mortality was performed prospectively from the end of the baseline period until date of death, emigration or end of follow‐up (31 December 2016), whichever occurred first.

### 2.4 Outcomes

Primary outcomes were OCS‐related morbidity and mortality. For morbidity, we considered the following OCS‐related complications that occurred during follow‐up: diabetes, osteoporosis, fractures, glaucoma, ischaemic heart disease, hypertension, psychiatric conditions, hypercholesterolaemia and sleep disorders. For mortality, we considered death from all causes during follow‐up, as well as cause‐specific mortality categorized using the underlying cause of death: respiratory related (ICD‐10: J as main cause of death), cardiovascular related (ICD‐10: I), cancer related (ICD‐10: C) and other.

### 2.5 Statistical analyses

Baseline characteristics were described as mean (standard deviation [SD]) for continuous variables and absolute and relative frequencies for categorical variables. For continuous variables the Kruskal‐Wallis rank sum test and for categorical variables the chi‐squared test were applied to test for difference between baseline OCS exposure groups. Regular OCS use during follow‐up was assessed through a sliding window of 365 days with patients collecting at least 1825 mg of prednisolone equivalent classified as regular OCS users. Cox proportional hazard models were applied to estimate hazard ratios (HR) with 95% confidence intervals (CI) of possible risk factors for becoming a regular OCS user for non‐OCS users at baseline. Age at asthma diagnosis, sex, asthma severity (GINA steps) and comorbidity (Charlson Comorbidity Index) were explored individually as well as in multivariable adjusted analysis. Potential OCS‐related morbidities were presented as age‐ and sex‐adjusted rates during follow‐up. Risk factors for all‐cause mortality were analysed using Cox proportional hazard regression, including baseline OCS exposure, age at asthma diagnosis, sex and comorbidity (Charlson index) explored individually and in mutually adjusted analysis. OCS use, risks of morbidities and cause‐specific mortality were analysed using Fine‐Gray regression accounting for competing risks of death.^18^, ^19^ Statistical analyses were performed using R version 3.3.2 (R foundation for Statistical Computing, Vienna, Austria, 2013).

## 3 RESULTS

**Figure 1:**
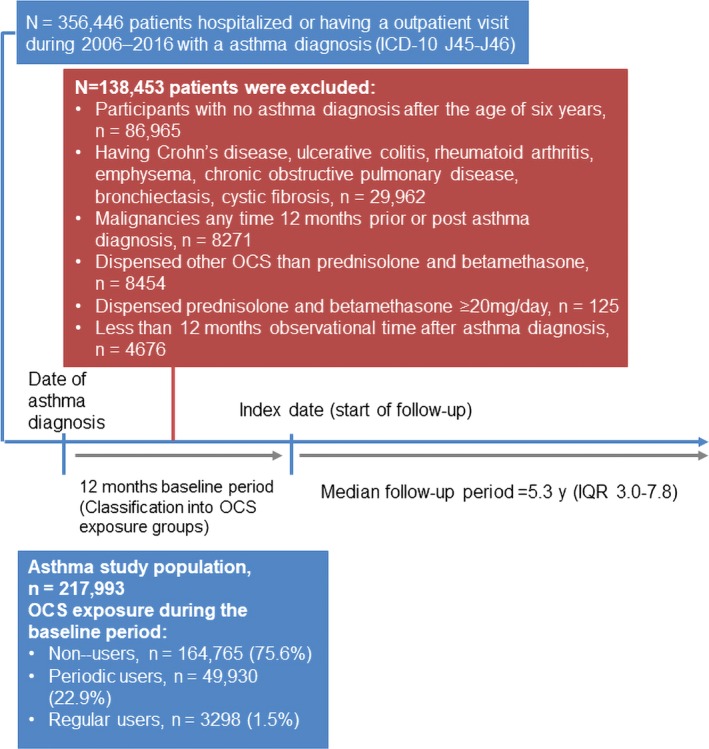
Flow chart of study population

Overall, 356 446 patients with a diagnosis of asthma and a collected asthma drug were identified, of whom 217 993 fulfilled the eligibility criteria during the observation period and were included in the study (Figure 1). The mean age of the study population was 33 years (SD 25.4), and 54% were women (Table 1). Median length of follow‐up was 5.3 years (interquartile range 3.0‐7.8 years; maximum 9.0 years) resulting in a total of 1 150 531 patient‐years of follow‐up*.*

**Table 1:** Baseline characteristics by use of oral corticosteroids at baseline

	Non‐OCS users n = 164 765	Periodic OCS users n = 49 930	Regular OCS users n = 3299	Total n = 217 994
Age (years, mean, SD)	30.7 (25.3)	40.1 (23.6)	61.3 (17.7)	33.3 (25.4)
Age group, n (%)				
<18	78 994 (47.9)	11 397 (22.8)	54 (1.6)	90 445 (41.5)
18‐40	29 149 (17.7)	13 229 (26.5)	327 (9.9)	42 705 (19.6)
40‐65	32 810 (19.9)	16 282 (32.6)	1376 (41.7)	50 468 (23.2)
>65	23 812 (14.5)	9022 (18.1)	1542 (46.7)	34 376 (15.8)
Women, n (%)	85 367 (51.8)	29 755 (59.6)	2042 (61.9)	117 164 (53.7)
Acute lower respiratory infections, n (%)	14 590 (8.9)	4618 (9.2)	347 (10.5)	19 555 (9.0)
Pneumonia, n (%)	14 179 (8.6)	5527 (11.1)	634 (19.2)	20 340 (9.3)
Nasal polyps, n (%)	3064 (1.9)	2631 (5.3)	418 (12.7)	6113 (2.8)
Chronic rhinitis, n (%)	2494 (1.5)	948 (1.9)	92 (2.8)	3534 (1.6)
Diabetes, n (%)	7912 (4.8)	2439 (4.9)	397 (12.0)	10 748 (4.9)
Ischaemic heart disease, n (%)	8510 (5.2)	2813 (5.6)	491 (14.9)	11 814 (5.4)
Heart failure, n (%)	4418 (2.7)	1565 (3.1)	367 (11.1)	6350 (2.9)
Stroke, n (%)	2688 (1.6)	766 (1.5)	131 (4.0)	3585 (1.6)
Osteoporosis, n (%)	2639 (1.6)	1089 (2.2)	294 (8.9)	4022 (1.8)
Glaucoma, n (%)	3429 (2.1)	1303 (2.6)	237 (7.2)	4969 (2.3)
Malignancies, n (%)	3171 (1.9)	1260 (2.5)	198 (6.0)	4629 (2.1)
Charlson Comorbidity Index, n (%)				
0‐1	146 134 (88.7)	43 705 (87.5)	1957 (59.3)	191 796 (88.0)
2	10 576 (6.4)	3477 (7.0)	680 (20.6)	14 733 (6.8)
3	5001 (3.0)	1762 (3.5)	354 (10.7)	7117 (3.3)
4+	3054 (1.9)	986 (2.0)	308 (9.3)	4348 (2.0)
Asthma medications, n (%)				
Inhaled corticosteroids (ICS)	83 504 (50.7)	2515 (51.1)	1487 (45.1)	110 506 (50.7)
Short‐acting ß2‐agonists	100 200 (60.8)	35 054 (70.2)	2113 (64.0)	137 367 (63.0)
Long‐acting ß2‐agonists (LABA)	13 248 (8.0)	7140 (14.3)	788 (23.9)	21176 (9.7)
Fixed ICS/LABA combination	50 082 (30.4)	22 887 (45.8)	1840 (55.8)	74 809 (34.3)
Fixed LABA/LAMA (long‐acting muscarinic antagonist)	597 (0.4)	1037 (2.1)	330 (10.0)	1964 (0.9)
Leukotriene modifiers	20 143 (12.2)	9722 (19.5)	899 (27.3)	30 764 (14.1)
LABA and LABA/LAMA without ICS	223 (0.1)	121 (0.1)	75 (0.2)	27 (0.8)
N‐acetylcysteine	10 279 (6.2)	8006 (16.0)	939 (28.5)	19 224 (8.8)
Long‐acting anticholinergics	3781 (2.3)	2713 (5.4)	431 (13.1)	6925 (3.2)
Adrenergics in combination with anticholinergics	597 (0.4)	1037 (2.1)	330 (10.0)	1964 (0.9)

Abbreviations: OCS, oral corticosteroids; SD, standard deviation.

At baseline, 1.5% of patients were classified as regular OCS users, 22.9% were periodic OCS users, and 75.6% were non‐OCS users (Table 1). Compared with periodic and non‐OCS users, patients on regular OCS treatment were older (mean age 61, vs 40 and 31 years, respectively). Women were more common for regular (62%) and periodic OCS users (60%) than in nonusers (52%). At baseline, monotherapy with inhaled corticosteroids (ICS) was more common in periodic and non‐OCS users than in regular users, whereas fixed ICS/long‐acting ß2‐agonists (LABA) combinations and fixed LABA/long‐acting muscarinic antagonist (LAMA) combinations were more commonly collected by regular OCS users. Regular OCS users also had a greater prevalence of nasal polyps, glaucoma, ischaemic heart disease, heart failure, malignancy and osteoporosis at baseline (Table 1). The periodic OCS users were older (mean age 40 vs 31 years), more often women (60% vs 52%), and showing a higher prevalence of pneumonia and nasal polys compared to the non‐OCS users. The periodic OCS users also collected SABA, LABA and fixed ICS/LABA combinations more frequently compared to the non‐OCS users.

### 3.1 Patterns of oral corticosteroids use

Each year, approximately 15% of the patients collected at least one OCS, which was relatively stable during the follow‐up period (Figure S1). Mean daily OCS dosage during follow‐up was 5.5 mg in the regular OCS group, 0.8 mg in the periodic group and 0.4 mg in patients who did not use OCS at baseline, (*P* < 0.001).

During follow‐up, more regular OCS users were hospitalized with asthma as the main reason for contact. Age‐adjusted hospitalization rate was 3.66 (3.22‐4.09) per 100‐patient‐years, compared with 1.48 (1.42‐1.54) in the periodic users and 0.51 (0.49‐0.53) in non‐OCS users. The proportion of patients seen in specialist care was similar between regular OCS users (41.3%) and periodic (42.2%) and nonusers (40.1%), although statistically significant different (*P* < 0.001).

**Figure 2:**
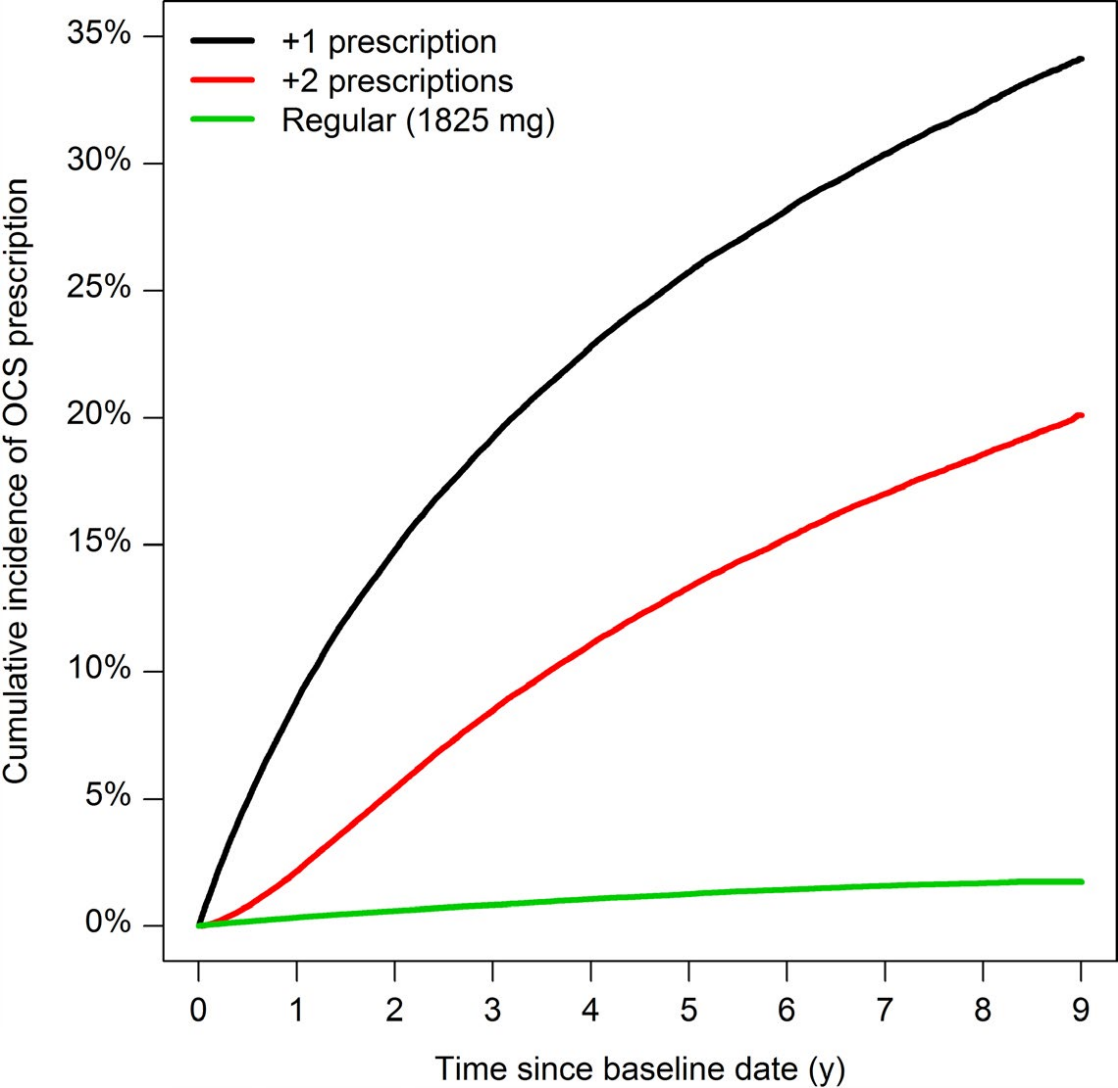
Cumulative risk of oral corticosteroid (OCS) exposure in patients without OCS use at baseline

After 9 years of follow‐up, the cumulative risk of dispensing at least one OCS prescription for non‐OCS users at baseline was 34.5% (95% CI, 34.2%‐34.9%), and 20.1% (95% CI, 19.8%‐20.4%) had collected two or more OCS prescriptions (Figure 2). Of nonusers at baseline, 1.3% had become regular OCS users during follow‐up. There was a pronounced increase in the cumulative incidence of OCS exposure in all age groups during follow‐up (Figure S2). Of the periodic OCS users at baseline, 6.1% became regular OCS users, whereas 59.3% remained periodic users throughout follow‐up. Of the regular OCS users at baseline, 25.8% became periodic users during follow‐up, whereas 64.9% remained regular users. In the total study population, 7316 patients (3.4%) became regular OCS users during the follow‐up period.

### 3.2 Factors associated with becoming a regular oral corticosteroids user

Of the non‐OCS users at baseline, older age at asthma diagnosis was the strongest independent risk factor for becoming a regular OCS user during follow‐up: people 65+ had a 19 times greater risk (HR 18.72, 95% CI 15.65‐22.39) compared with people <18 years (Table 2). Additional risk factors were greater GINA asthma severity steps, increased Charlson Comorbidity Index and to some degree female sex.

**Table 2:** Risk factors for becoming a regular OCS user among non‐OCS users at baseline

Variable	Crude		Adjusted model	
	HR (95% CI)	*P*‐Value	HR (95% CI)	*P*‐Value
Age category				
<18	1.00		1.00	
18‐40	4.40 (3.63‐5.32)		3.47 (2.84‐4.25)	
40‐65	12.76 (10.85‐15.01)		9.23 (7.75‐10.99)	
>65	27.76 (23.67‐32.56)	<0.001	18.72 (15.65‐22.39)	<0.001
Female sex	1.84 (1.68‐2.01)	<0.001	1.19 (1.08‐1.30)	<0.001
GINA step				
Step 1	0.98 (0.78‐1.22)		1.10 (0.88‐1.38)	
Step 2	1.31 (1.09‐1.58)		1.21 (1.01‐1.45)	
Step 3	1.82 (1.53‐2.17)		1.41 (1.19‐1.68)	
Step 4	2.60 (2.19‐3.09)	<0.001	2.03 (1.71‐2.42)	<0.001
Charlson comorbidity index				
0‐1	1.00		1.00	
2	3.85 (3.42‐4.34)		1.16 (1.02‐1.31)	
3	3.96 (3.36‐4.68)		1.16 (0.97‐1.37)	
4+	5.21 (4.25‐6.37)	<0.001	1.34 (1.09‐1.65)	<0.001

Note

Cox regression of time to first regular use of oral corticosteroid (OCS) prescription among patients without OCS use at baseline, unadjusted or mutually adjusted for the factors in the model.

Abbreviations: CI, confidence interval; GINA, Global Asthma Initiative, and greater steps reflect greater asthma severity; HR, hazard ratio; OCS, oral corticosteroids.

### 3.3 Oral corticosteroids use, morbidity and mortality

More patients died and were censored due to development of potential OCS‐treated diseases and malignancy in the regular OCS group than the other OCS groups (Figure S3).

**Figure 3:**
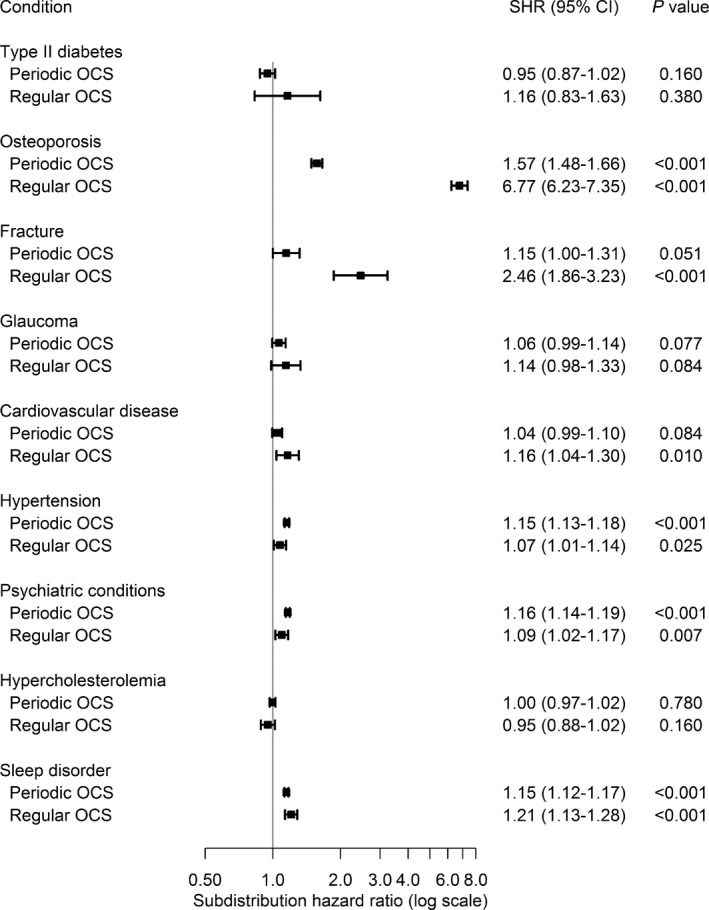
Risk of oral corticosteroid (OCS)‐related morbidities compared to OCS nonusers. Estimates were analysed as sub‐distribution hazard rates (SHR) using Fine‐Gray regression adjusted for age and sex, accounting for the competing risk of death

The risk of developing a potential OCS‐related morbidity was greater for regular OCS users compared to periodic and non‐OCS users (Figure 3). In competing risk regression adjusted for age and sex, the risk of osteoporosis was almost 7‐fold greater for regular OCS users (sub‐distribution hazard ratio [SHR] = 6.77, 95% CI = 6.23‐7.35) and almost 2‐fold greater for periodic OCS users (SHR 1.57, 95% CI 1.48‐1.66) compared to non‐OCS users. Corresponding relative risk of fractures was 2.46 (95% CI 1.86‐3.23) for regular users and 1.15 (95% CI 1.00‐1.31) for periodic users.

**Figure 4:**
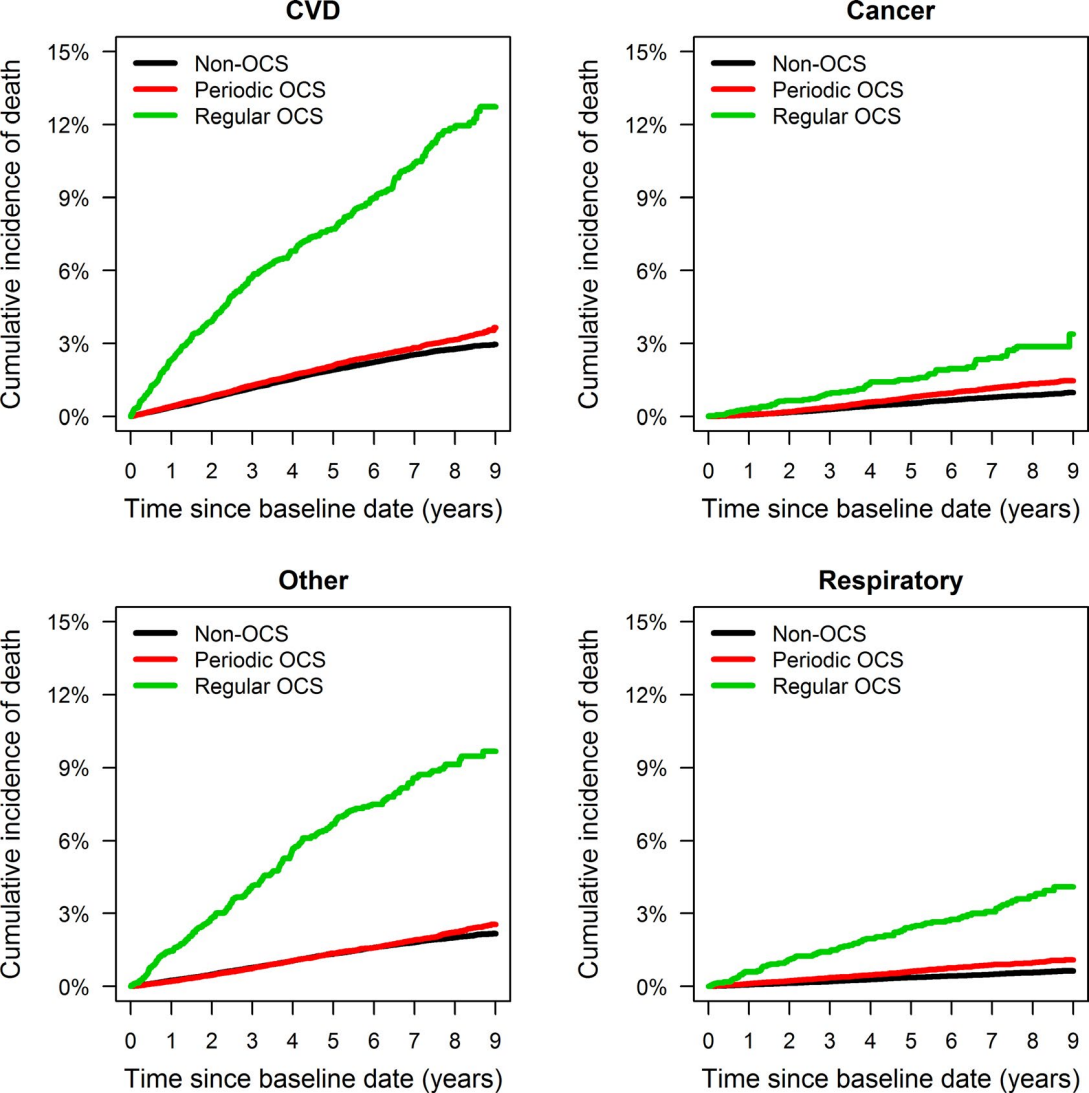
Cumulative incidence of cause‐specific death stratified by oral corticosteroid (OCS) exposure during baseline period [Color figure can be viewed at http://www.wileyonlinelibrary.com]

During follow‐up, 21.0% of the asthma patients in the regular OCS users group died compared with 5.5% of the periodic users, and 4.5% of the non‐OCS users. The most common cause of death was cardiovascular disease, followed by cancer and respiratory causes (Figure 4). Other causes were primarily deaths related to dementia, Alzheimer’s disease, diabetes and sepsis. Regular OCS users had an overall greater risk of death than nonusers, HR 1.34 (95% CI 1.24‐1.45, *P* < 0.001) adjusted for age, sex and Charlson Comorbidity Index (Table 3).

**Table 3:** Multivariable cox regression model for all‐cause mortality in asthma patients

	Crude	Adjusted
	HR (95% CI)	HR (95% CI)
OCS exposure baseline period		
No exposure	1.00	1.00
Periodic exposure	1.21 (1.16‐1.27)	0.95 (0.91‐0.99)
Regular exposure	5.00 (4.63‐5.41)	1.34 (1.24‐1.45)

* Mutual adjusted for age, sex, and Charlson Comorbidity Index

## 4 DISCUSSION

In this nationwide population‐based longitudinal study of patients identified in secondary care, almost one in seven asthma patients used OCS each year. Among patients with no OCS use at baseline, 26% collected at least one OCS prescription during the median 5.5 years observation period and 1.3% became regular OCS users. However, 3.4% of all patients were regular OCS users for at least 1 year during the study period. Key determinants of regular OCS use were age at asthma diagnosis, asthma severity and increased comorbidity. Regular OCS use was associated with markedly increased risks of developing OCS‐related morbidities and with greater all‐cause and cause‐specific mortality, particularly cardiovascular mortality.

The present national linked data set is uniquely placed to describe the patterns of OCS use in the whole asthma population identified in secondary care in Sweden and related outcomes. The nationwide analysis ensured that the challenges with selection bias of patients because of inclusion of selected hospitals, regions or healthcare insurance systems are reduced and enhances the generalizability of the study findings.

The limitation that some OCS use could relate to other conditions than asthma was addressed by excluding patients treated with OCS medications or high‐dosage treatment not commonly used for asthma, and patients with conditions for which OCS may be prescribed. In addition, if patients were diagnosed with these conditions during follow‐up, they were censored at that time. A challenge was to decide whether to include or exclud patients with closely related asthma co‐morbidities (such as severe rhinitis, rhinosinusitis with or without nasal polyps or bronchiectasis), that may also be indications for OCS treatment in severe asthma patients. As our intention was to describe the OCS use directly related to asthma, we utilized a conservative approach and excluded patients with bronchiectasis in order not to overestimate the OCS use that is directly related to asthma. We did, however, include rhinitis and rhinosinusitis as regular OCS treatment is seldom use in these conditions. Furthermore, there is always a risk that some of the patients have a COPD component together with their asthma diagnosis. However, we have excluded patients with COPD at baseline, and patients were censored if/when a COPD diagnosis was recorded during follow‐up. In addition, few patients were treated with typical COPD therapy alone at baseline (LABA as monotherapy without ICS [1.0%] or fixed LABA/LAMA monotherapy without ICS [<0.1%]), thus the impact of a potential COPD component is limited.

A potential source of underreporting of OCS use could be the exclusion of other OCS medications than the ones we have included in the analyses. However, only 1.3% of the patients collected other types of OCS during follow‐up, thus the impact of the exclusion of other OCS medications can be regarded as limited (Figure S3). Another potential limitation was that OCS use was based only on collected prescriptions, which, as in most pharmacoepidemiologic investigations, might not fully reflect patients’ actual medication use. As a registry data‐based analysis, the study relied on ICD‐10 codes for assessment of the morbidity and mortality study outcomes, so the possibility of coding errors cannot be completely ruled out. However, validation studies have reported high correlation between data in the Swedish National Patient Register and diagnoses in medical records.^11^, ^12^

There are few comparable studies having a nationwide population perspective on OCS use in an asthma population. Some reports describe the overall OCS use for patients in primary care, and there are reports on determinants for OCS use and OCS‐related adverse events in different asthma populations.^9^, ^20^, ^21^, ^22^ A Swedish primary care study reported a lower proportion of asthma patients being regular OCS users for at least 1 year (1.4%) compared with our study (3.4%).^19^ A possible explanation for the differences in estimates of regular OCS use between our study and the previous study may be because the patients in our study emanated from secondary care, which may represent a more severe asthma population.

Our results indicated that, annually, almost one in seven asthma patients had used OCS, which was stable over the 10‐year study period. Of the non‐OCS users at baseline, a cumulative risk of 35% and 20% for prescribing one or more than one OCS prescriptions during follow‐up was observed. OCS use is considered to be a marker of uncontrolled asthma, either as exacerbation treatment or as a regular maintenance treatment to prevent disease deterioration.^3^ The present findings indicate that in our population, a significant percentage of asthma patients experience a disease severity that requires OCS to manage their symptoms. Interestingly, the percentage of asthma patients falling into this category remained rather stable throughout the 10‐year study period. These findings are in line with other studies with more selected asthma populations.^23^ Our findings on the factors associated with regular OCS use (asthma severity level, older age and greater comorbidity burden) are in keeping with the findings from previous studies.^6^, ^8^, ^22^ However, in our data set we did not have access to data on other potential important determinants for OCS use in asthma patients, such as smoking.

A large proportion of the regular OCS users continued with regular therapy during the 10‐year study period, with a mean daily dosage of 5.5 mg, a dosage which in many countries is considered to be a low maintenance dosage and in line with GINA recommendations.^8^ However, also this low maintenance dosage was associated with increased risk of developing potentially OCS‐related diseases. We observed a marked increased risk of developing osteoporosis among the regular OCS users when compared to periodic or nonusers, and all other OCS‐related diseases followed the same pattern. Our findings of increased risk of OCS‐related adverse events are consistent with previous reports.^7^, ^22^ In addition, also the periodic users had an increased risk of OCS‐related diseases compared to the non‐OCS users, further highlighting the negative consequences of increased OCS use.

Compared with periodic and non‐OCS use, regular use was associated with greater all‐cause mortality. This observation persisted even after adjusting for sex and age. There were more deaths among the regular OCS users compared to non‐OCS users particularly related to cardiovascular disease, malignancies, other reasons (mainly dementia related) and respiratory diseases. We cannot rule out that patients with severe and terminal illness might have been prescribed regular OCS therapy without a related diagnosis in a hospital setting. However, we think that it is unlikely that such patients would have received regular OCS therapy over a full calendar year. Our findings of increased risk of deaths among regular OCS users when compared to nonusers agree with reports from other previous studies.^9^, ^24^

The present findings have several potential clinical implications. The GINA guidelines recommend assessment, life style counselling and regular monitoring of patients with asthma who receive OCS as maintenance therapy with treatment lengths for 3 or more months.^2^ New biologic treatment options have recently been introduced for the treatment of severe asthma patients.^25^, ^26^, ^27^, ^28^, ^29^ Studies have demonstrated that biologics can reduce the number of exacerbations significantly compared with placebo, especially in patients on treatment with OCS, a patient group generating a high burden to health care and health‐related cost for society.^10^, ^30^, ^31^ These new treatments may allow patients to reduce the dosage and potentially stop their OCS therapy.

The GINA guidelines also recommend that patients with severe asthma should be managed by asthma specialists.^2^ Interestingly, in our study, the percentage of patients having been seen in outpatient secondary care because of asthma during the observation period was approximately the same (40%) in the three OCS use groups, despite regular OCS users having a more severe asthma with more asthma‐related hospitalizations. This finding may suggest that severe asthma patients might be an overlooked patient population, which is supported by a previous Swedish report that only 1 of 5 severe asthma patients in primary care was referred to secondary care.^3^ Furthermore, patients in Swedish primary care suffering from frequent asthma exacerbations do not seem to be identified and managed in accordance with guideline recommendations, indicating a room for improvement.^3^

In conclusion, the present findings demonstrate that OCS therapy is still a substantial part of current asthma management for a high percentage of patients and that regular OCS use is associated with severe side effects and mortality risk. The study indicates that there is a need for use of other treatment options for patients with severe asthma who are using regular OCS.

## CONFLICT OF INTERESTS

PH and GT are employed by AstraZeneca. FW is employed at Statisticon for which AstraZeneca is a client. ME, BN and CJ report no conflict of interest relevant to this article.

## AUTHORS’ CONTRIBUTIONS

Data collection was performed by JB. Statistical analysis was conducted by FW and ME. Analysis, interpretation and drafting of the manuscript were conducted by ME and PH and in cooperation with the other authors. All authors approved the manuscript before submission.

## ETHICAL APPROVAL

The study was approved by the Stockholm regional ethics committee (registration number 2017/4:2). The linkage of registers data was approved and performed by the Swedish National Board of Health and Welfare. Patients do not need to give consent for use of public register data in Sweden.

## CONSENT FOR PUBLICATION

All authors read and approved the final manuscript. All authors gave consent to publish these data.

## Data Availability

The data set supporting the conclusions of this article can be available upon request.
