# Supplementary material for: Oral corticosteroid use, morbidity and mortality in asthma: A nationwide prospective cohort study in Sweden
Source: Allergy. 2019 Jun 11;74(11):2181–90. doi: 10.1111/all.13874 (PMC6899917; doi:10.1111/all.13874)
Supplement: Supplementary file 1 [file ALL-74-2181-s001.docx]

**Supplementary data**

**Figure 1.** Annual proportion of patients that collected at least one prescription of medication after baseline period**.**

**
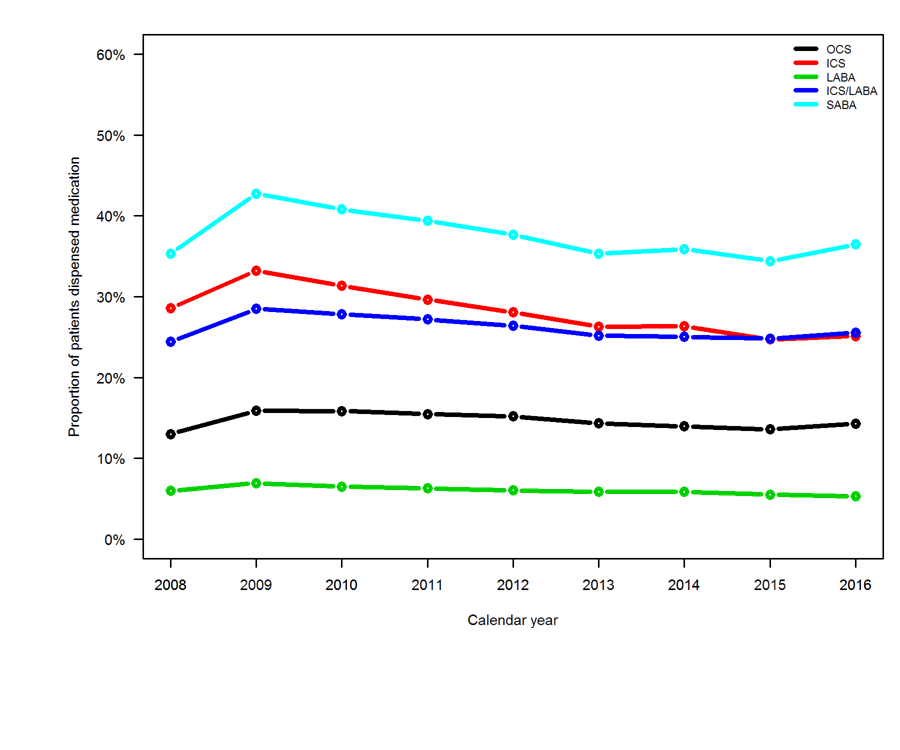
Figure 2.** Cumulative incidence of prescription for OCS (left panels) and regular OCS exposure (right panels) after baseline period among non-OCS exposed during baseline period


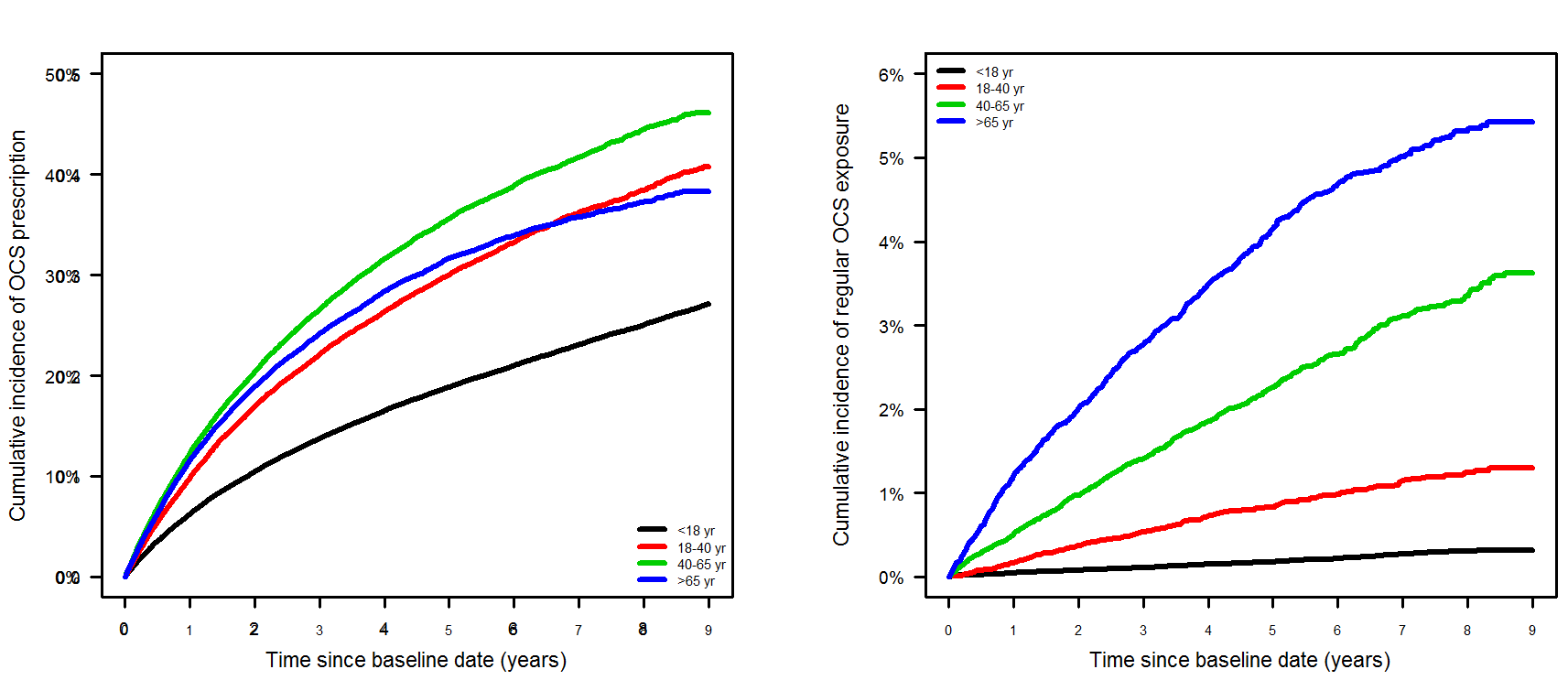


**Figure 3.** Flow-chart of asthma study population during follow-up

**
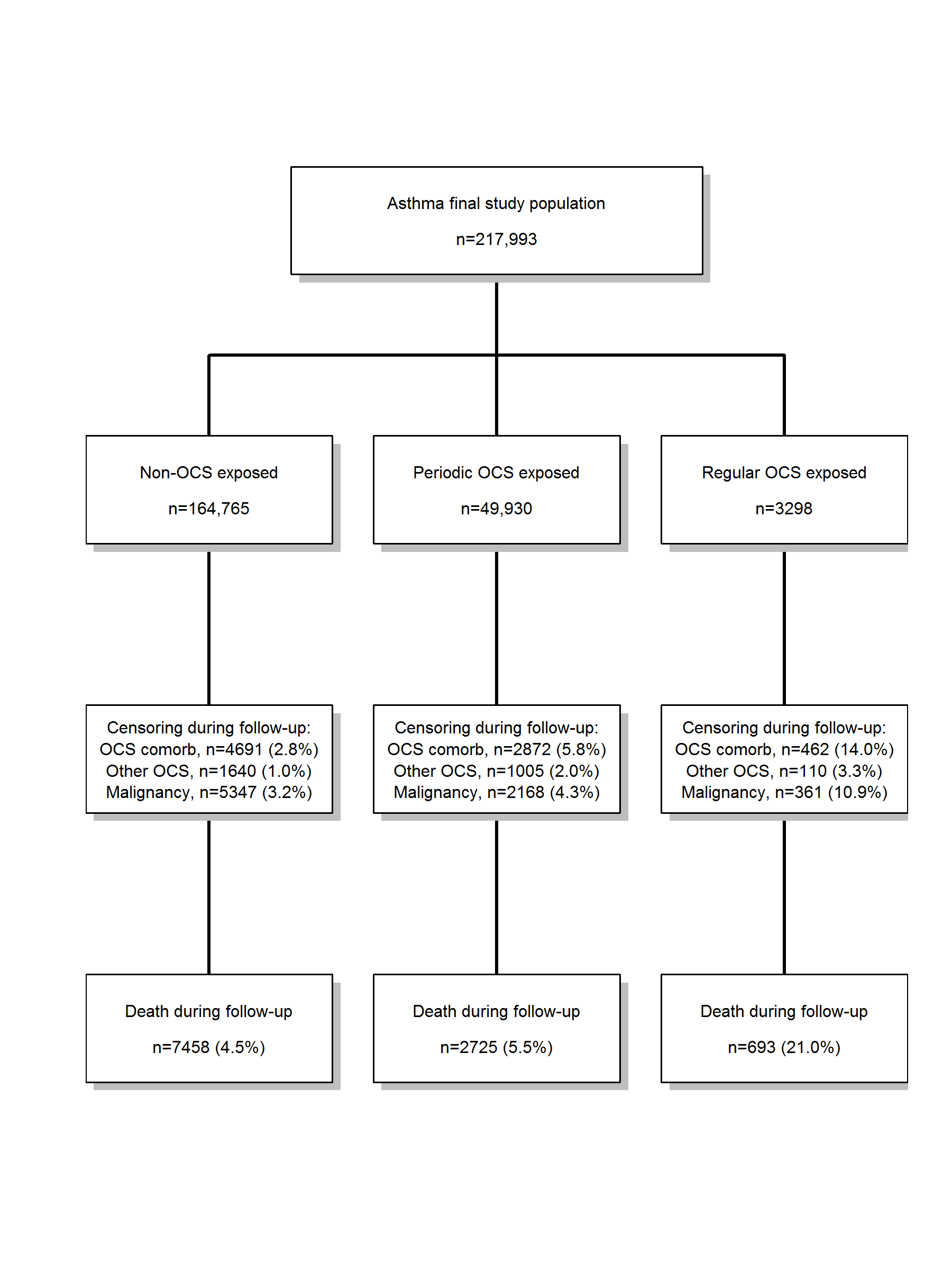
**
